# Special Issue “Recent Advances in Nanoparticles in Molecular Biology”

**DOI:** 10.3390/ijms26136321

**Published:** 2025-06-30

**Authors:** Maciej Monedeiro-Milanowski

**Affiliations:** Centre for Modern Interdisciplinary Technologies, Nicolaus Copernicus University in Torun, 87-100 Torun, Poland; milanowski.maciej@gmail.com

Nanoparticles are small particles that range between 1 and 100 nanometers in size. Their significant applications cover various fields such as the environment, agriculture, food, biotechnology, biomedicine, and medicines, for example, for the treatment of wastewater, environmental monitoring, use as functional food additives, and use as antimicrobial agents [1,2]. Considering their nanoscale characteristics, various nanoparticles (e.g., metals and metal oxides) can be collocated with biomolecules, such as proteins, nucleic acids, and polysaccharides, to utilize the inherent characteristics of both systems, resulting in novel biomolecule–nanoparticle hybrids.

This Special Issue “Recent Advances in Nanoparticles in Molecular Biology” showcases the most recent reports on the synthesis, characterization, and functionalization of nanoparticles in molecular biology. In this Special Issue, readers will find eight contributions, namely six original works and two reviews, which may be accessed here: https://www.mdpi.com/journal/ijms/special_issues/J274BFYTAW (accessed on 24 June 2025).

The first review [3] provides an excellent survey of the most recent low-cost, small-sized zinc oxide nanoparticles (ZnO NPs) employed for seed nano-priming. Particularly, ZnO NPs are used in the field of agriculture and food industries as fertilizers and micronutrients in mainly zinc-depleted soils. The role of ZnO NPs in seed nano-priming is to alleviate plants’ abiotic and biotic stress.

The authors explain the effects of seed priming through several mechanisms, such as the stimulation and improvement of seed metabolic rate, vigor index, and seedling characteristics, while reducing toxic metal accumulation through increased antioxidant enzyme activity and decreased oxidative stress. A major focus is on using ZnO NPs to combat abiotic stresses such as drought, salinity, and heavy metal or arsenic contamination. For example, rice seeds treated with ZnO NPs showed improved drought tolerance, while a durum wheat variety (*Triticum durum* L.), *Sorghum bicolor*, and rapeseed varieties benefited under salt stress. ZnO NP priming also reduced heavy metal uptake (Cu, Co, Pb, and Cd) in *Zea mays* L. seeds, *Basella alba* L., and popular varieties of rice seeds (Xiangyaxiangzhan and Yuxiangyouzhan). In arsenic-contaminated soils, ZnO NP treatment significantly mitigated toxicity in *Vigna mungo* (L.) Hepper plants.

Another section highlights ZnO NPs as a tool for managing soil- and seedborne pathogens. Sustainable crop production, in terms of yields and quality, may be ensured using the simultaneous action of ZnO NPs and endophytic bacteria. For example, ZnO NPs with the beneficial fungus *Trichoderma harzianum* were used for nano-priming chickpea seeds (*Cicer arietinum* L.). Additionally, ZnO NPs with *R. leguminosarum* reduced nodulation, blight disease indices, galling, and nematode population.

The administration of ZnO NPs in pre-storage treatments showed great potential in seed invigoration, quality improvement, and the amelioration of the physiological parameters of seeds. These benefits were demonstrated in two different-aged lots of green gram seeds and chickpea seeds.

The authors show several examples of enhanced production, improved seed germination profiles, and boosted seedling developments due to the application of ZnO NPs. It was confirmed for the priming of wheat (*Triticum indicum* and *Triticum aestivum*); rice (*Oryza sativa* L.); and widespread crops such as tomato seeds (*Lycopersicon esculentum* Mill), cotton seeds, and faba beans (*Vicia faba* L.).

Finally, the work provides an outlook on the future directions and shortcomings of ZnO NP priming. The benefits of ZnO NPs priming are very dose-dependent. The authors suggest conducting comparative studies to correlate the inherent action mechanism with ZnO NP characteristics. They advocate for the creation of a database with ZnO NP-related parameters of cultivation, the standardization of ZnO NP synthesis, and the development of ZnO NPs with simple and low-cost coatings to yield target crops.

The second review [4] highlights the recent applications of nanomaterials in the treatment of pancreatic-related diseases, including pancreatic cancer, which emerges as a global health challenge.

The first topic that was raised was the discussion of the diverse nature of common nanomaterials used in the treatment of pancreatic cancer, pancreatitis, and diabetes. Four groups of nanomaterials have been defined, namely inorganic, carbon-based, polymeric nanomaterials, and liposomal nanoparticles.

Nanoparticles are shown to work with certain biological effects or release their payload, such as drugs or cytokines, to exert therapeutic effects. They can increase the accumulation of antitumor drugs in pancreatic tumor sites due to the enhanced permeability and retention effect of tumor tissues. Additionally, they act as carriers of various bioactive molecules, such as anti-inflammatory drugs or immune modulators, effectively altering the immune microenvironment of the pancreas. Nanomaterials can be administered into the body via different routes which significantly affect their interactions with the pancreas. However, convenient oral administration needs to be improved in terms of bioavailability and pancreatic targeting of nanomaterials. Intravenous injections are one of the primary delivery routes to the pancreas. They benefit from bypassing the degradative effects of the gastrointestinal tract. However, nanomaterials are easily captured by the reticuloendothelial system; hence, improved specificity of the targeting molecules and avoiding immune system recognition are crucial. As another technique, an intraperitoneal injection is a direct, precise, and dose-effective method. Unfortunately, it is disadvantaged by complex procedures and injection site infections.

The third section provides a detailed overview of how nanomaterials are applied in the treatment of pancreatic cancer, pancreatitis, and diabetes. The authors also point out their most important limitations. Pancreatic cancer-targeted nanomaterials are optimized to operate in three areas of action. They improve drug delivery efficiency, for example TAB004-NPs, FAD-Dox, and KRAS-siRNA NP, but they might be unvalidated or unstable in in vivo tests and require large-scale production. GDNDs, CONPs, and Col-TNP enhance the efficacy of radiotherapy by, e.g., absorbing incident light or sensitizing pancreatic cancer cells to RT, but they are challenging to produce and still need to be further studied. GFNPS-GEM and cRGD-GdIO-DTX improve the integration of diagnosis and treatment; however, their drug loading efficiency can be insufficient, and cytotoxicity may increase at prolonged and high doses of exposure. Nanomaterials used in pancreatitis treatment have been developed as drug-loaded carriers (e.g., MU and apigenin-loaded PLGA nanoparticles) but their insufficient targeting, controllability, and stability might pose issues. The enhancement of anti-inflammatory effects (e.g., selenium nanoparticles and CO-HbVs) is obtained through the regulation of macrophage and neutrophil activity or by lessening the developed pancreatic injury, but this is hampered by dose control issues, limited light penetration depth, and increased toxicity. Nanomaterials such as LR-SSVA, AT-CC, and LA-PC inhibit pancreatic fibrosis through high selectivity, strong targeting, and a dual-action approach but suffer from stability and control issues and complicated preparation processes. Diabetes treatment can be facilitated by improved insulin delivery efficiency, enhanced insulin sensitivity, and the protection of islet cells. Diabetes-targeted (MOF) NU-1000 and CV@INS@ALG, as well as functionalized fullerenes and silver and zinc oxide nanoparticles provide improved insulin absorption efficiency and drug delivery stability and exhibit antioxidant and anti-inflammatory properties. High production costs, not well-elucidated mechanisms, and long-term storage stability often limit their application. Moreover, nanomaterials can safeguard islet cells. For example, metallic nanoparticles (Au, Ag, and Cu) and metallic oxide (ZnO) have excellent antioxidant and anti-inflammatory effects and are good drug carriers. MECA79-anti-CD3-NPs and Ac4ManNAz NPs are highly targeted and precise. Their shortcomings arise from long-term safety and efficacy, toxicity control, and unclear metabolic and clearance pathways.

Finally, the authors look forward to the prospects of nanomaterials. A personalized treatment stemming from the integration of combined genomics, proteomics, and nanotechnology can lead to achieving true precision medicine. Additionally, the precision treatment of pancreatic diseases can benefit from the fusion of gene editing systems with nanotechnology, optimization of nanocarriers to enhance the delivery system, and the concept of nanoparticle-assisted delivery of probiotics or prebiotics to the gut.

This Special Issue also includes six published papers that offer examples and a comprehensive overview of the current use of nanoparticles in molecular biology. The most important findings from these original works are discussed below:

1. Antioxidant Iron Oxide Nanoparticles: Their Biocompatibility and Bioactive Properties. The authors prepared gallic acid-functionalized iron oxide nanoparticles (GA-IONPs) through a mild process in deionized water and tested them for potential antioxidant and biocompatibility properties. GA-IONPs were confirmed via TEM, HR TEM, FT IR, and SQUID analyses. The cell viability remained above 95% against concentrations up to 0.5 mg/mL; therefore, they exhibited no cytotoxicity. Under the same concentration, the fluorescence intensity decreased by approximately 70%; hence, GA-IONPs functioned as antioxidants and acted as ROS scavengers, causing the inhibition of H_2_O_2_ activity. No genotoxic effects associated with IONPs were reported. The observation of tetraspanins’ (CD9, CD81, and CD63) tendency to be present in exosomes released from H_2_O_2_-stimulated and GA-IONP-treated human dermal papilla cells revealed outstanding cell protection behavior. GA-IONPs appeared to be synthesized in an eco-friendly manner without harsh conditions or high temperatures [5]. These properties highlight the potential of GA-IONPs for safe application in therapeutic or diagnostic nanomedicine through a combination of environmentally friendly synthesis and enhanced stability, strong antioxidant effects, and low cytotoxicity in human cells [6].
2. Self-Entrapment of Antimicrobial Peptides in Silica Particles for Stable and Effective Antimicrobial Peptide Delivery System. Researchers aimed to enhance the stability and safety of antimicrobial peptides (AMPs) applicable to effective drug–device combination products. They wanted to overcome their two limitations, namely their sensitivity to proteolytic degradation and toxicity to mammalian cells. The shortest human cathelicidin LL37-derived peptide, KR12, was fused with a cell penetrating peptide (CPP) derived from human immunodeficiency virus TAT to form CPP-KR12. Then, the authors constructed CPP-KR12@Si—a new product derived from forming silica particles with self-entrapped CPP-KR12 peptide using biomimetic silica precipitability. The antibacterial activity of CPP-KR12 increased approximately 8-fold against *E*. *coli* and approximately 30-fold against *P*. *aeruginosa*, hence indicating improved bactericidal effect against both Gram-negative and Gram-positive bacteria. CPP-KR12 can enter the cell without damage to the membrane when applied at low concentrations; however, at higher concentrations it might cause disruption of the membrane, leading to cell lysis [7]. Moreover, CPP-KR12 entirely stopped DNA migration at a concentration of 4 µM, demonstrating an affinity for intracellular DNA. Considering the above, the antibacterial activity of CPP-KR12 is manifested mainly through its increased membrane permeability, high affinity for binding to DNA, as well as the ROS generation effect of KR12. Both KR12 and CPP-KR12 showed the capability to undergo silica formation. Encapsulated CPP KR12@Si showed improved resistance to trypsin digestion versus KR12, KR12@Si, and CPP-KR12. This provides the benefits of a controlled release rate and potential prolonged circulation of AMPs in the bloodstream. CPP-KR12@Si notably reduced hemolytic activity against sheep red blood cells and cytotoxicity toward Raw264.7 macrophage cells. Finally, the constructed CPP-KR12@Si-coated bone graft substitutes were a profitable result of a biocompatible method for creating silica hybrids by mimicking the positively charged peptides involved in silica deposition. The new product was shown to be effective against *E*. *coli* infections, hence showing promise in future drug–device projects [8].
3. A Multifunctionalized Potyvirus-Derived Nanoparticle That Targets and Internalizes into Cancer Cells. The authors successfully purified, for the first time [9,10], and engineered viral nanoparticles (VNPs) derived from turnip mosaic virus (TuMV) through functionalization using the Z domain of staphylococcal Protein A via gene fusion. TuMV exhibits a high affinity for immunoglobulins G (IgG). Cetuximab, an epidermal growth factor receptor (EGFR) inhibitor, was chosen to demonstrate the versatility of TuMV VNP by constructing a fluorescent nanoplatform to mark tumoral cells from the Cal33 line of a tongue squamous cell carcinoma. Virus-like particles (VLPs) are structures that mimic the appearance of viruses but lack any infectious ability, as they are formed by self-assembled capsid proteins. Constructs of TuMV with VLPs, instead of containing other VNPs, are twice as long (around 720 nm) and offer more binding sites to the IgGs. TEM and Western blot/ELISA analyses confirmed the successful binding of the VLP–Z domain constructs to cetuximab. A colorimetric assay revealed that none of the constructs affected cell viability. Confocal microscopy and flow cytometry assays demonstrated that VLP–cetuximab–Cy5.5 complexes from functionalization A (cetuximab first) bound selectively to Cal33 cells (overexpressing EGFR) but not to EGFR-negative THP1. Moreover, this complex bound to the cells to a greater extent than cetuximab-absent VLP–Cy5.5. Additionally, VLP–cetuximab–Cy5.5 appeared to enter the cells after 24 h of incubation and tended to accumulate in a perinuclear location. The main limitation of the study was believed to be the absence of fluorescence in the construct in which Cy5.5 was conjugated first, followed by the addition of cetuximab to the VLP [11].
4. Nanoremediation and Antioxidant Potential of Biogenic Silver Nanoparticles Synthesized Using Leucena’s Leaves, Stem, and Fruits. Silver nanoparticles (AgNPs) were synthesized using an aqueous extract from the leaves, stem, and fruits of *Leucaena leucocephala* (Leucena), which is a highly invasive species [12]. AgNPs were characterized through multiple techniques, namely UV–vis, TEM, EDS, SDL, XPS, XRD, and zeta potential. TEM analysis revealed that most of the nanoparticles had quasi-spherical shapes. The researchers optimized the Leucena extraction procedure to obtain the highest possible amounts of phenolic acids due to their important role in the green or biogenic synthesis of metallic nanoparticles since they induce the reduction of metal ions and act as capping agents. Phenolic acids are also known for their antioxidant activity [13]. The results indicated that the maximized amounts of phenolic acids were extracted by maintaining a temperature of 80 °C for 20 min for the leaves and stem and 30 min for the fruits. All three AgNP types effectively remediated synthetic dyes pollution in aqueous samples, namely methylene blue and tartrazine. The one synthesized from the leaves was the most efficient. Additionally, the leaf-derived AgNPs exhibited superior antioxidant activity that surpassed that of butylated hydroxytoluene. The authors stated that the usage of this proposed extraction protocol from the leaves of an invasive species can provide the formation of AgNPs which efficiently remediate organic environmental pollution [14].
5. Networked Cluster Formation via Trigonal Lipid Modules for Augmented Ex Vivo NK Cell Priming. Conventional cytokine-based priming of natural killer (NK) cells can cause the deactivation of biological signaling molecules and subsequent insufficient maturation of the cell population during mass cultivation processes. The goal of the study was to overcome such limitations by introducing a cytokine-free priming method using a novel trigonal-linker (TL) module. This new construct results from the conjugation of 1,2-distearoyl-sn-glycero-3-phosphoethanolamine-N-[amino(polyethylene glycol)-2000] (DSPE-PEG-NH_2_) with trimesic acid (TMA) and thus the formation of (DSPE-PEG-NH)_3_-T. The DSPE lipid anchor could be inserted into NK cell membranes via hydrophobic interaction between DSPE lipid and the lipid bilayers of NK cell surfaces. The reduced viability of NK cells was not reported up to 200 µg/mL of TL modules. The investigation of IFN-γ secretion in TLNK cells after 6 h and 24 h of spheroid formation revealed that TLNK spheroid exhibited enhanced levels of IFN-γ secretion at both time intervals [15]. Therefore, this approach improved inter-membrane contact within the NK cell population and increased frequent signal exchange. The authors concluded that this newly synthesized TL module effectively initiated NK cell clustering without any inhibition in proliferation and other intrinsic cellular properties. Additionally, this approach enables augmented NK cell activation without the need for additional supplementary cytokine cocktails. Finally, the developed method offers a scalable alternative to conventional cytokine-dependent NK cell expansion—potentially improving efficacy and reliability in NK cell-based immunotherapies [16].
6. Solid Lipid Nanoparticles Delivering a DNA Vaccine Encoding *Helicobacter pylori* Urease A Subunit: Immune Analyses before and after a Mouse Model of Infection. In the study, novel solid lipid particles (SLN-A) containing the adjuvant lipid monophosphoryl lipid A were synthesized and characterized using DLS and TEM. SLN-A(H) particles had an average diameter of 98.0 (±6.9) nm with an average zeta potential of 55.9 (±3.7) mV, and they appeared as a mixture of spherical and cuboid-shaped structures. The average particle diameter was found to be 78.1 ± 41.3 nm. These lipid carriers efficiently complexed with a DNA vaccine encoding the urease A subunit of *Helicobacter pylori*, forming “lipoplex-A”. In a mouse model of *H*. *pylori* infection, the lipoplex-A nanoparticles were used to immunize mice, and the resultant immune responses were analyzed. A prime with lipoplex-A and a boost with soluble UreA protein produced high IgG1 levels, and two doses of lipoplex-A induced high levels of the IgG2c antibody, thus indicating flexibility in shaping Th1/Th2 responses. An analysis of immune cell populations from mouse stomach tissue revealed that both the lipoplex-A vaccine boosted with protein and lipoplex-A induced significant levels of CD4^+^ T cells [17]. However, mice vaccinated with the lipoplex-UreA vaccine did not have reduced levels of colonization compared to the infection control (PBS) or empty lipoplex groups. These findings highlight the system’s potential for delivering DNA vaccine-encoded antigens to effectively stimulate immune responses and indicate its capacity to tailor the nature of those responses [18].

The Guest Editor is grateful to all authors who submitted their work and supported this collection and to the *International Journal of Molecular Sciences* staff, whose help was invaluable for the success of this editorial project. I hope that this Special Issue helps to enhance the current knowledge on recent applications of nanoparticles in molecular biology and provides valuable insights that can guide future research on their emerging functions.

## References

[1] Altammar K.A. (2023). A Review on Nanoparticles: Characteristics, Synthesis, Applications, and Challenges. Front. Microbiol..

[2] Rajesh Kumar M., Joice Sophia P., Kumar D., Gong C. (2018). Nanoparticles as Precious Stones in the Crown of Modern Molecular Biology. Trends in Insect Molecular Biology and Biotechnology.

[3] Donia D.T., Carbone M. (2023). Seed Priming with Zinc Oxide Nanoparticles to Enhance Crop Tolerance to Environmental Stresses. Int. J. Mol. Sci..

[4] Ma J., Li X., Wang C. (2024). The Application of Nanomaterials in the Treatment of Pancreatic-Related Diseases. Int. J. Mol. Sci..

[5] Lee J., Morita M., Takemura K., Park E.Y. (2018). A Multi-Functional Gold/Iron-Oxide Nanoparticle-CNT Hybrid Nanomaterial as Virus DNA Sensing Platform. Biosens. Bioelectron..

[6] Lee J., Lee J.-H., Lee S.-Y., Park S.A., Kim J.H., Hwang D., Kim K.A., Kim H.S. (2023). Antioxidant Iron Oxide Nanoparticles: Their Biocompatibility and Bioactive Properties. Int. J. Mol. Sci..

[7] Sato H., Feix J.B. (2006). Peptide–Membrane Interactions and Mechanisms of Membrane Destruction by Amphipathic α-Helical Antimicrobial Peptides. Biochim. Biophys. Acta-Biomembr..

[8] Ki M.-R., Kim S.H., Park T.I., Pack S.P. (2023). Self-Entrapment of Antimicrobial Peptides in Silica Particles for Stable and Effective Antimicrobial Peptide Delivery System. Int. J. Mol. Sci..

[9] Cuesta R., Yuste-Calvo C., Gil-Cartón D., Sánchez F., Ponz F., Valle M. (2019). Structure of Turnip Mosaic Virus and Its Viral-like Particles. Sci. Rep..

[10] Truchado D.A., Rincón S., Zurita L., Sánchez F., Ponz F. (2023). Isopeptide Bonding In Planta Allows Functionalization of Elongated Flexuous Proteinaceous Viral Nanoparticles, Including Non-Viable Constructs by Other Means. Viruses.

[11] Truchado D.A., Juárez-Molina M., Rincón S., Zurita L., Tomé-Amat J., Lorz C., Ponz F. (2024). A Multifunctionalized Potyvirus-Derived Nanoparticle That Targets and Internalizes into Cancer Cells. Int. J. Mol. Sci..

[12] de Sousa Machado M.T., Drummond J.A., Barreto C.G. (2020). Leucaena Leucocephala (Lam.) de Wit in Brazil: History of an Invasive Plant. Estud. Ibero-Am..

[13] Barakat A.Z., Hamed A.R., Bassuiny R.I., Abdel-Aty A.M., Mohamed S.A. (2020). Date Palm and Saw Palmetto Seeds Functional Properties: Antioxidant, Anti-Inflammatory and Antimicrobial Activities. J. Food Meas. Charact..

[14] Silva C.S., Tonelli F.M.P., Delgado V.M.S., Lourenço V.d.O., Pinto G.d.C., Azevedo L.S., Lima L.A.R.d.S., Furtado C.A., Ferreira D.R.C., Tonelli F.C.P. (2024). Nanoremediation and Antioxidant Potential of Biogenic Silver Nanoparticles Synthesized Using Leucena’s Leaves, Stem, and Fruits. Int. J. Mol. Sci..

[15] Lin Q., Rong L., Jia X., Li R., Yu B., Hu J., Luo X., Badea S.R., Xu C., Fu G. (2021). IFN-γ-Dependent NK Cell Activation Is Essential to Metastasis Suppression by Engineered Salmonella. Nat. Commun..

[16] Park J., Kim S., Jangid A.K., Park H.W., Kim K. (2024). Networked Cluster Formation via Trigonal Lipid Modules for Augmented Ex Vivo NK Cell Priming. Int. J. Mol. Sci..

[17] Skakic I., Francis J., Dekiwadia C., Aibinu I., Huq M., Taki A., Walduck A., Smooker P. (2023). An Evaluation of Urease A Subunit Nanocapsules as a Vaccine in a Mouse Model of Helicobacter Pylori Infection. Vaccines.

[18] Francis J.E., Skakic I., Majumdar D., Taki A.C., Shukla R., Walduck A., Smooker P.M. (2024). Solid Lipid Nanoparticles Delivering a DNA Vaccine Encoding Helicobacter Pylori Urease A Subunit: Immune Analyses before and after a Mouse Model of Infection. Int. J. Mol. Sci..

